# Clinical, Radiographic, and Return-to-Sport Outcomes After MINAR Reconstruction for Acute High-Grade Acromioclavicular Joint Separation in Recreational Football Players

**DOI:** 10.3390/jcm15207907

**Published:** 2026-10-13

**Authors:** Aydogan Aşkın, Vugar Nabi, Fatih Duygun, Ömer Faruk Kılıçaslan, Ibrahim Etli, Ali Bülent Baz, Akın Bilgin, Caner Yıldırım

**Affiliations:** Department of Orthopaedics and Traumatology, Antalya Training and Research Hospital, University of Health Science, 07100 Antalya, Türkiye; aydoganaskin1@gmail.com (A.A.); fduygun2000@yahoo.com (F.D.); kilicaslanfaruk@hotmail.com (Ö.F.K.); ietli@hotmail.com (I.E.); abulentb@gmail.com (A.B.B.); akinbilgin@gmail.com (A.B.); cnryldrm98@gmail.com (C.Y.)

**Keywords:** acromioclavicular joint separation, Rockwood classification, MINAR reconstruction, coracoclavicular fixation, vertical reduction loss, return to sport, football, retrospective analysis

## Abstract

**Background:** High-grade acromioclavicular (AC) joint separations are clinically relevant injuries in contact sports, yet data specific to recreational football players remain limited. This study evaluated clinical recovery, maintenance of vertical coracoclavicular reduction, and return-to-sport outcomes after minimally invasive acromioclavicular reconstruction (MINAR) in recreational players with acute Rockwood type IV-V AC joint separation. **Methods:** This single-center retrospective analysis included 49 male recreational football players who sustained acute, isolated, unilateral Rockwood type IV or V acromioclavicular (AC) joint separation during football participation between January 2015 and December 2023. All patients underwent surgery within 7 days of injury using the same MINAR construct and implant platform, and all procedures were performed by the same experienced surgical team. Postoperative rehabilitation was supervised by the institutional physiotherapy department, generally three times weekly, with progression individualized according to clinical recovery. The primary outcome was the change in Constant score from preoperative assessment to 24 months. Secondary outcomes included Taft, American Shoulder and Elbow Surgeons (ASES), and visual analog scale (VAS) pain scores; minimal clinically important difference (MCID) achievement; coracoclavicular (CC) distance; return to unrestricted match play; return to preinjury football level; and exploratory comparisons according to vertical reduction status. Coracoclavicular distance was measured independently by three blinded observers, and the mean of the three measurements was used for the radiographic analyses. Radiographic loss of vertical reduction was defined a priori as a 24-month injured-to-contralateral CCD ratio greater than 1.50. Because only five patients met this criterion, comparisons according to vertical reduction status were considered exploratory and descriptive. **Results:** The mean age was 35.1 ± 6.2 years, the mean body mass index was 27.9 ± 5.2 kg/m^2^, and the mean follow-up was 48.6 ± 14.5 months. Forty-one patients had Rockwood type V injuries and eight had type IV injuries. All clinical scales improved over time (Friedman *p* < 0.001). The Constant score increased from 29.9 ± 6.0 preoperatively to 94.5 ± 3.8 at 24 months. Mean preoperative-to-24-month improvements were 64.6 points in Constant score, 6.11 points in Taft score, and 43.3 points in ASES score, while VAS pain decreased by 6.50 points. All 49 patients exceeded the prespecified Constant-score MCID threshold, and 47 of 49 patients (95.9%) exceeded the Taft-score MCID threshold. Return to unrestricted match play occurred in all patients at a mean of 239.9 ± 43.5 days, and 44 patients (89.8%) returned to their preinjury match level. Five patients met the radiographic criterion for loss of vertical reduction (10.2%; 95% CI, 4.4–21.8). In exploratory unadjusted comparisons, these five patients returned to unrestricted match play later than patients who maintained vertical reduction (332.4 ± 35.6 vs. 229.4 ± 29.8 days; unadjusted mean difference, 103.0 days; 95% CI, 59.5–146.5), and none returned to the preinjury football level (0/5 vs. 44/44). These sparse subgroup comparisons were interpreted descriptively. **Conclusions**: In this cohort of male recreational football players with acute Rockwood type IV-V acromioclavicular joint separation, MINAR reconstruction was followed by substantial improvement in shoulder function, minimal residual pain, and a high rate of return to football. Maintenance of vertical reduction and return-to-sport outcomes showed a clinically notable pattern in exploratory unadjusted analyses; however, the reduction-loss subgroup comprised only five patients, and patient-level chronology did not uniformly support a temporal pathway from reduction loss to delayed return. These findings should therefore be regarded as hypothesis-generating rather than predictive or causal.

## 1. Introduction

Acromioclavicular (AC) joint separation is a common shoulder-girdle injury in contact and collision sports, with potentially relevant implications for pain, shoulder function, training continuity, and return to play [1,2]. In football, AC joint injuries are frequently related to direct contact or fall mechanisms, and available data from collegiate and elite cohorts indicate that these injuries may affect player availability and sports performance after return [1,2]. However, the existing sport-specific literature is weighted toward elite or professional athletes, whereas recreational football players—who represent a large proportion of patients encountered in routine orthopedic practice—remain less well characterized [2,3,4,5,6].

Although surgical treatment of high-grade AC joint separation generally yields favorable clinical outcomes, interpretation of the return-to-sport literature is limited by substantial heterogeneity in Rockwood grade, the timing of surgery, fixation technique, rehabilitation strategy, and return-to-play definitions [3,4,5,6,7]. In many studies, return to sport is reported primarily as a time-based or binary outcome, while the quality of recovery, return to preinjury level, residual symptoms, and maintenance of vertical reduction are less consistently integrated into outcome assessment [5,6]. This distinction is clinically important because a patient may return to match participation without fully recovering preinjury function or confidence. Therefore, evaluating patient-reported outcomes, pain, radiographic vertical stability, and football-specific return milestones in the same cohort may offer a more clinically meaningful assessment of recovery after high-grade AC reconstruction.

Sports participation should not be regarded as uniform musculoskeletal exposure, because the repetitive loads, movement patterns, and physical demands imposed on athletes differ considerably across disciplines and may lead to sport-specific functional and anatomical adaptations. This is particularly evident around the shoulder girdle. Chiaramonte et al., for example, demonstrated greater external rotation of the dominant shoulder in adolescent volleyball players and position-related changes in glenohumeral morphology, illustrating how repeated sport-specific loading may alter joint characteristics without necessarily representing pathological instability [8]. The biomechanical environment of football is different, combining intermittent high-intensity activity with direct contact, falls, fatigue, and variable playing conditions. In a systematic review, Cardoso-Marinho et al. found that football players commonly identified insufficient strength and physical fitness, fatigue, excessive training load, and playing-surface characteristics among factors perceived to influence injury risk [9]. Although these observations do not establish a direct relationship with AC joint separation, they emphasize that injury susceptibility and subsequent recovery should be interpreted within the specific biomechanical and exposure profile of each sport. This perspective is particularly relevant when evaluating return to football after an injury to the shoulder girdle, where successful recovery requires more than restoration of pain-free function during routine daily activities.

Within this sport-specific context, recreational football players may also differ from professional athletes in technical proficiency, neuromuscular preparedness, exposure to structured injury-prevention programs, rehabilitation access, and supervised load management [10,11,12,13]. These factors do not imply that recreational players necessarily sustain more severe AC injuries, but they may plausibly influence postoperative recovery and sport-specific readiness. Accordingly, this study aimed to evaluate clinical, radiographic, and return-to-sport outcomes in male recreational football players with acute, isolated, unilateral Rockwood type IV-V AC joint separation treated within 7 days using the same MINAR construct and implant platform, by the same experienced surgical team, and under an institutional rehabilitation protocol. We hypothesized that patients would show substantial functional improvement and a high rate of return to football. We additionally explored whether loss of postoperative vertical reduction co-occurred with delayed match-level return and less complete recovery to the preinjury level of sport, while recognizing that the small number of reduction-loss events precluded causal, confirmatory, or definitive predictive inference.

## 2. Materials and Methods

### 2.1. Study Design and Setting

This single-center retrospective analysis was performed by reviewing routine clinical records and radiographic data from patients treated surgically for acute AC joint separation between January 2015 and December 2023. This study was prepared in accordance with the Strengthening the Reporting of Observational Studies in Epidemiology (STROBE) statement [14].

### 2.2. Participants

All consecutive patients who underwent surgery for AC joint separation at the study institution during the study period were screened for eligibility. Recreational football participation was defined as regular non-professional small-sided football played on artificial-turf pitches, without a professional contract. Patients were included if they had regular match participation; sustained the injury during football participation; had an acute, isolated, unilateral Rockwood type IV or V AC joint separation; underwent surgery within 7 days of injury; were treated by the same surgical team using the same MINAR construct, implant platform, and surgical principles; participated in the department-supervised postoperative rehabilitation pathway; and had available clinical and radiographic follow-up data at 24 months. No football position-based exclusion criterion was applied, and all eligible recreational players were included.

### 2.3. Eligibility Criteria

Exclusion criteria were previous injury or surgery involving the same shoulder; concomitant clavicle, scapular, or proximal humeral fracture; neurovascular injury; bilateral injury; polytrauma preventing participation in the postoperative rehabilitation pathway; need for additional surgery for another shoulder pathology during the same operative session; and insufficient follow-up data. Rockwood type IV and V injuries were included because these high-grade injuries are generally considered operative lesions in active patients.

### 2.4. Data Collection

Demographic, perioperative, clinical, radiographic, complication, and return-to-sport data were extracted from institutional clinical records, operative notes, imaging archives, and follow-up records. Recorded baseline variables included age, body mass index (BMI), smoking status, injured side, dominant-side involvement, Rockwood classification, time from injury to surgery, follow-up duration, and radiographic CC measurements. Missing data were handled by complete-case analysis; all variables required for the primary and secondary analyses were available in the final cohort.

### 2.5. Imaging Protocol and Radiographic Assessment

Diagnosis was based on trauma history, physical examination, true anteroposterior and bilateral Zanca radiographs, and computed tomography (CT) imaging obtained according to the institutional imaging protocol. Bilateral focused radiographs were used to assess vertical displacement [15,16]. During the study period, dynamic horizontal stress radiographs, including Alexander and cross-body adduction views, were not routinely obtained. Instead, axial CT images and multiplanar or three-dimensional reconstructions were used to assess the static anteroposterior relationship between the distal clavicle and the acromion, particularly when a Rockwood type IV injury was suspected. Because CT was obtained in the supine, non-weight-bearing position, it could identify static posterior displacement but could not quantify dynamic horizontal translation under functional loading [16,17]. Coracoclavicular distance (CCD) was measured digitally using the institutional picture archiving and communication system and was defined as the shortest perpendicular distance between the superior cortex of the coracoid and the inferior cortex of the clavicle. The contralateral CCD measured on the baseline bilateral radiograph was used as the reference value for serial injured-side measurements. Injured-side CCD was recorded preoperatively, on the first postoperative radiograph, and at 3, 6, 9, 12, and 24 months. Measurements were performed independently by three blinded investigators, and the mean of the three observer measurements was used for the primary radiographic analyses. The injured-to-contralateral CCD ratio was calculated at each follow-up assessment. Radiographic loss of vertical reduction was defined a priori as a 24-month injured-to-contralateral mean CCD ratio greater than 1.50, corresponding to an injured-side CCD more than 50% greater than that of the contralateral side. Serial CCD measurements therefore assessed vertical CC alignment only and should not be interpreted as an assessment of overall three-dimensional AC joint stability. The first scheduled radiograph at which the ratio exceeded 1.50 was recorded as the first detection of loss of vertical reduction. Because imaging was performed at predefined follow-up intervals, this represents interval-censored detection rather than the exact biological onset of displacement. The 1.50 threshold was not selected by optimizing associations with the clinical outcomes of the present cohort. An injured-to-uninjured CCD ratio greater than 150% has previously been used to define reduction loss after AC joint fixation, while related studies have used a 50% displacement or subluxation criterion to identify substantial radiographic failure after AC joint stabilization [16,17,18]. Routine MRI was not obtained because the diagnosis and operative indication for otherwise isolated acute high-grade AC joint separation were established clinically and radiographically. MRI was reserved for clinical findings suggestive of associated glenohumeral or soft-tissue pathology. Consequently, clinically silent associated intra-articular lesions could not be completely excluded [15,16,18].

### 2.6. Surgical Technique

All procedures were performed by the same experienced surgical team using the standard MINAR (Karl Storz SE & Co. KG, Tuttlingen, Germany; with FLIPPTACK extracortical fixation buttons) technique. Through a limited superior approach, a dedicated aiming guide was used to place a guidewire centrally at the base of the coracoid, with the corresponding clavicular tunnel positioned directly above the coracoid base. After fluoroscopic confirmation, 4.5 mm coracoid and clavicular tunnels were created, and a two-button suspensory construct was passed, with the inferior button being deployed beneath the coracoid and the superior button being seated on the clavicle after reduction. Guide position, tunnel placement, reduction, and final button position were checked fluoroscopically at the relevant operative steps. No graft augmentation or additional ligament repair was performed, and the AC capsule was neither exposed nor repaired [19,20].

### 2.7. Rehabilitation and Follow-Up Protocol

Postoperative rehabilitation was supervised by the hospital physiotherapy department, generally with three sessions per week. Rehabilitation followed a broad phase-based pathway comprising initial sling-based protection, progressive passive and active-assisted motion, restoration of active range of motion, strengthening, and subsequent sport-specific exercises. Progression was individualized by the treating physiotherapist according to pain, range of motion, strength, and clinical recovery; therefore, an identical week-by-week progression or standardized return-to-sport clearance protocol was not applied to every patient. Quantitative session-level rehabilitation adherence was not consistently available for retrospective analysis. The exact duration of sling use was not uniformly retrievable from the final retrospective dataset and was therefore not analyzed as a standardized exposure. Return to unrestricted match play was defined as the first patient-reported calendar date, documented in the routine clinical follow-up record, on which the participant resumed full, unrestricted recreational football match participation without shoulder-related self-modification. Formal team-based medical clearance was generally unavailable in this recreational cohort; accordingly, this variable represents observed return to participation rather than standardized medical clearance. Return to the preinjury football level was defined as patient-reported resumption of the same participation frequency and perceived level as before injury [5,6].

### 2.8. Outcome Measures

The primary clinical outcome was the change in Constant score between the preoperative assessment and 24-month follow-up. The Constant–Murley score is a multidimensional shoulder score assessing pain, activities of daily living, range of motion, and strength [21,22]. The original 12-point Taft score was used as an AC joint-specific secondary outcome, with higher scores indicating a better clinical and radiographic result [23]. The ASES score assessed pain and daily function from the patient perspective [24]. The VAS pain score was used to quantify pain severity [25]. Clinical scores were recorded preoperatively and at 3, 6, 12, and 24 months. Clinically meaningful improvement was evaluated using the AC joint-specific, anchor-based thresholds reported by Stein et al. after stabilization of acute high-grade AC joint injuries: 16.63 points for the Constant score and 2.87 points for the Taft score. For reporting clarity, these thresholds were rounded to 16.6 and 2.9 points, respectively [26].

### 2.9. Statistical Analysis

Continuous variables were assessed using histograms, Q–Q plots, and the Shapiro–Wilk test. Data were summarized as means ± standard deviations or medians with interquartile ranges, as appropriate. Categorical variables were reported as counts and percentages. Repeated clinical scores across five time points were analyzed using the Friedman test. Preoperative-to-24-month changes were analyzed using the paired-samples t-test or Wilcoxon signed-rank test, depending on distributional characteristics. Main change estimates were reported with 95% confidence intervals. Cohen’s dz was calculated for parametric paired analyses and interpreted as a standardized within-patient change measure rather than a between-treatment effect. MCID achievement and return-to-sport proportions were reported with Wilson 95% confidence intervals. Interobserver reliability of CCD measurements was evaluated separately at each radiographic assessment using a two-way random-effects, absolute-agreement intraclass correlation coefficient. Both the reliability of a single observer measurement [ICC(2,1)] and the reliability of the mean of three observer measurements [ICC(2,3)] were calculated and reported with 95% confidence intervals [27]. Because only five patients met the radiographic loss-of-vertical-reduction criterion, comparisons according to reduction status were considered exploratory. Baseline characteristics according to reduction status were presented descriptively without inferential hypothesis testing. Continuous outcomes were compared using the Mann–Whitney U test and were summarized using both means ± standard deviations and medians with interquartile ranges where relevant. For time to unrestricted match play, the unadjusted between-group mean difference was additionally reported with a Welch 95% confidence interval. Categorical outcomes were compared using Fisher’s exact test, and the absolute risk difference for return to preinjury level was reported with a Newcombe–Wilson 95% confidence interval. No multivariable regression or prediction model was fitted for reduction status. Because several outcomes were examined in a sparse subgroup, the corresponding *p*-values were interpreted descriptively and were not considered confirmatory evidence of independent prediction or causality. A post hoc threshold-sensitivity analysis repeated the 24-month reduction-loss classification using injured-to-contralateral CCD-ratio thresholds of greater than 1.25, 1.30, 1.40, and 1.50. Two-sided *p* < 0.05 was considered statistically significant. Analyses were performed using IBM SPSS Statistics for Windows, version 26.0 (IBM Corp., Armonk, NY, USA), with formula-based calculations used where required for Cohen’s dz and interval estimates.

### 2.10. Ethical Considerations

This study was conducted in accordance with the Declaration of Helsinki and was approved by the University of Health Sciences, Antalya Training and Research Hospital Ethics Committee (approval number: IRB-2020-117; approval date: 4 June 2020). Written informed consent for the use of clinical and radiographic data was obtained from all participating patients or their legal representatives according to institutional regulations. The preoperative surgical informed-consent process included permission for anonymized clinical and radiographic data to be used for scientific purposes.

## 3. Results

### 3.1. Cohort Characteristics

The final analysis included 49 male recreational football players. The mean age was 35.1 ± 6.2 years, the mean BMI was 27.9 ± 5.2 kg/m^2^, and the mean follow-up duration was 48.6 ± 14.5 months. Surgery was performed at a mean of 2.8 ± 1.1 days after injury. Forty-one patients were classified as Rockwood type V and eight as Rockwood type IV. Twenty-seven patients were smokers, 32 had right-sided injuries, and the dominant side was injured in 31 patients. No reoperation, infection, or hardware irritation was recorded (Table 1).

Radiographic loss of vertical reduction was identified in five patients (10.2%; 95% CI, 4.4–21.8). Descriptive baseline characteristics according to vertical reduction status are provided in Table 2.

**Figure 1:**
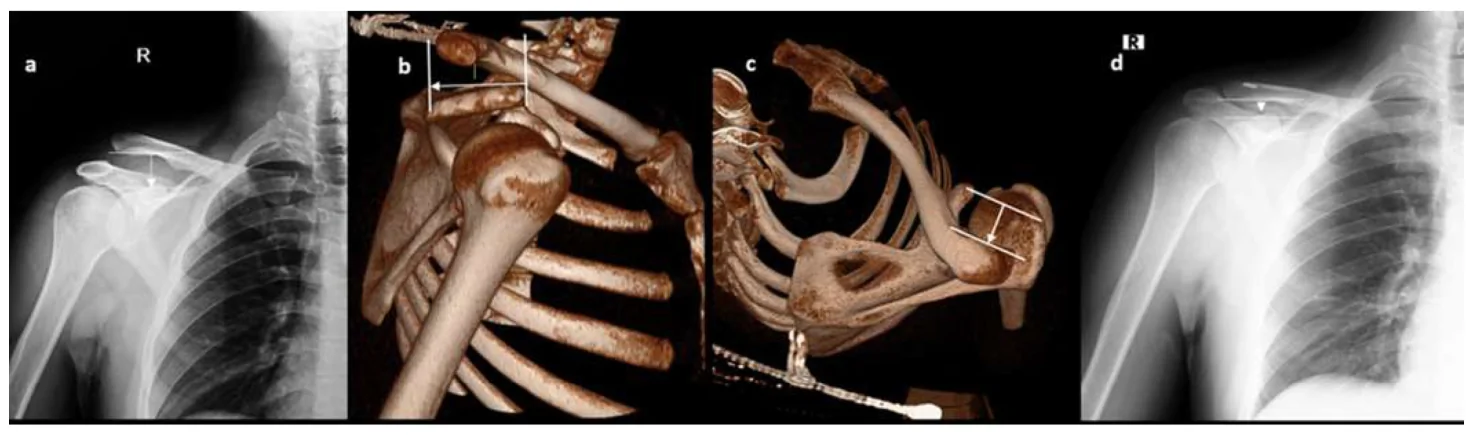
Rockwood type IV acromioclavicular joint separation. Preoperative anteroposterior radiograph (**a**) and three-dimensional computed tomography reconstructions (**b**,**c**) demonstrate posterior displacement of the distal clavicle relative to the acromion, consistent with a Rockwood type IV injury. Because horizontal instability may not be adequately appreciated in standard radiographs, axial CT reconstructions may improve diagnostic assessment. Postoperative radiograph (**d**) demonstrates restoration of AC alignment after MINAR fixation.

**Figure 2:**
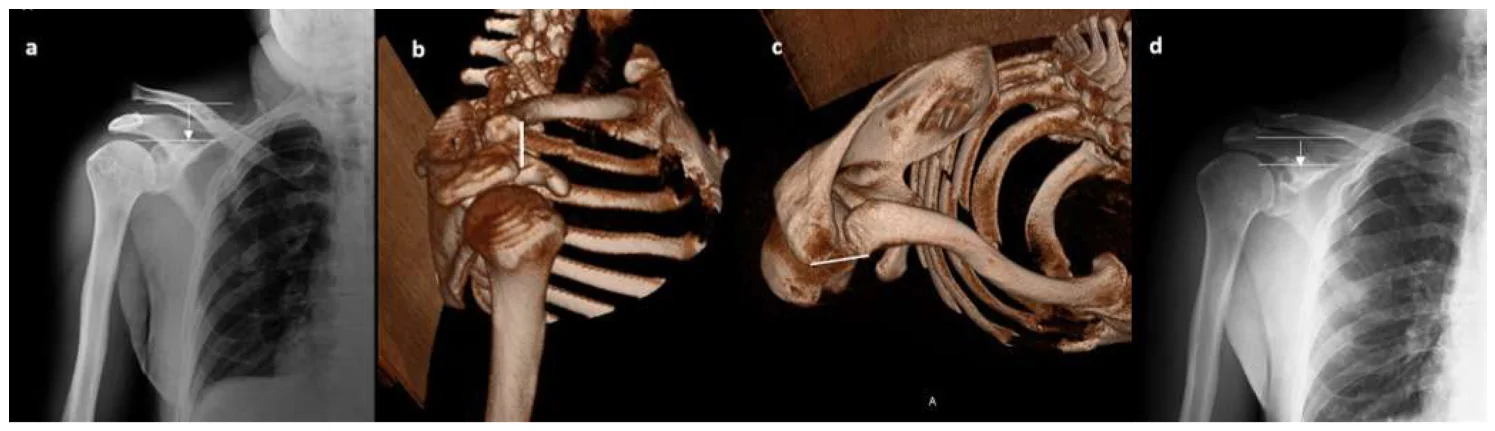
Rockwood type V acromioclavicular joint separation. Preoperative anteroposterior radiograph (**a**) and three-dimensional CT reconstructions (**b**,**c**) demonstrate marked superior displacement of the distal clavicle with a substantial widening of the CC interval, consistent with Rockwood type V injury. Posterior translation is absent. Postoperative radiograph (**d**) demonstrates restoration of the CC relationship after MINAR fixation.

**Figure 3:**
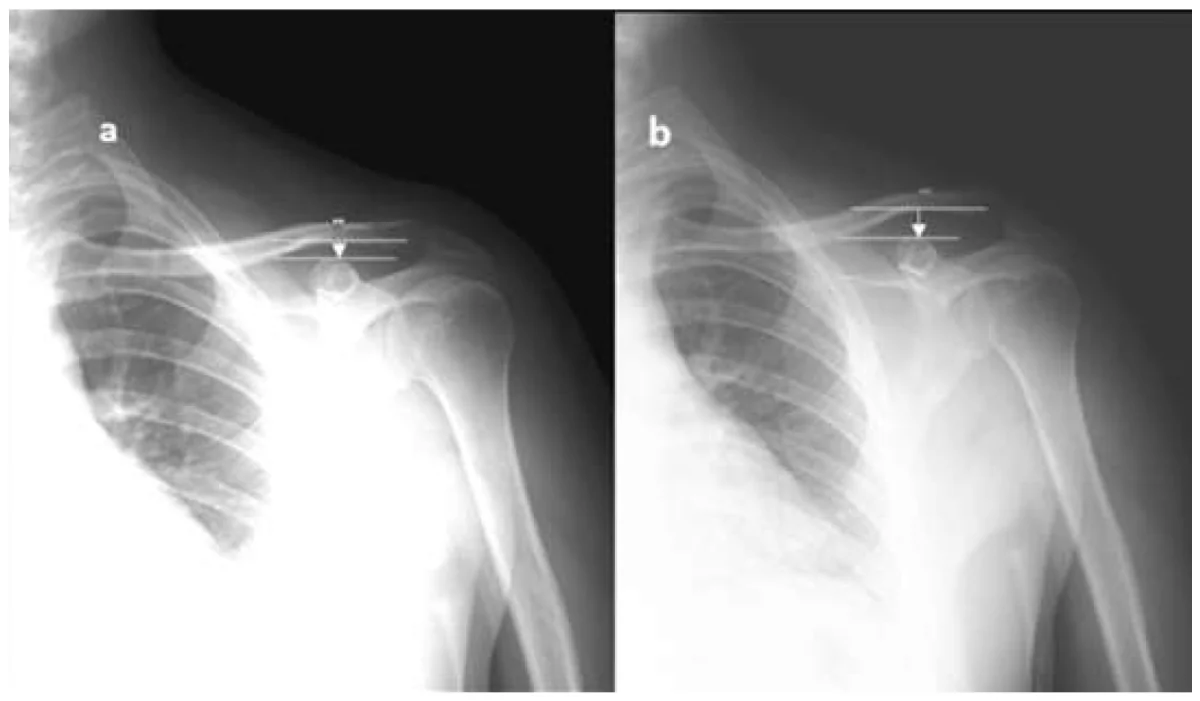
Loss of reduction after MINAR fixation. Immediate postoperative anteroposterior radiograph (**a**) demonstrates anatomic reduction of the AC joint after MINAR fixation with restoration of the CC distance. Follow-up anteroposterior radiograph (**b**) shows postoperative loss of reduction, characterized by superior migration of the distal clavicle and increased CC distance while the implant remains in situ.

Representative imaging examples of Rockwood type IV and type V injuries and loss of reduction are shown in Figure 1, Figure 2 and Figure 3, respectively. No patient met the vertical reduction-loss criterion on the first postoperative radiograph or at 3 or 6 months. Four patients first exceeded the prespecified ratio threshold at the scheduled 9-month examination, localizing threshold crossing to the interval between the 6- and 9-month assessments. The remaining patient had a CCD ratio of 1.249 at 12 months and first exceeded the criterion at 24 months (ratio, 1.501), localizing threshold crossing to the interval between the 12- and 24-month assessments. Patient-level chronology is presented in Table 3.

### 3.2. Clinical Outcome Trajectory

All clinical scales improved significantly over time, with Friedman *p* < 0.001 for each scale. The Constant score increased from 29.9 ± 6.0 preoperatively to 89.9 ± 6.4 at 3 months and 94.5 ± 3.8 at 24 months. The Taft score increased from 4.7 ± 0.7 preoperatively to 8.6 ± 0.2 at 3 months, 9.9 ± 0.8 at 6 months, 10.4 ± 0.8 at 12 months, and 10.8 ± 0.8 at 24 months. ASES scores also improved over time, while VAS pain decreased from 6.8 ± 1.5 preoperatively to 0.3 ± 0.5 at 24 months. Most clinical improvement occurred within the first 3 to 6 months, followed by smaller favorable consolidation between 6 and 24 months. Longitudinal clinical outcomes are summarized in Table 4.

### 3.3. Clinically Meaningful Improvement and Effect Size

From preoperative assessment to 24 months, the mean Constant score increased by 64.60 ± 7.47 points (95% CI, 62.45–66.75), the Taft score increased by 6.11 ± 1.26 points (95% CI, 5.74–6.47), and the ASES score increased by 43.32 ± 7.09 points (95% CI, 41.28–45.35), while VAS pain decreased by 6.50 ± 1.60 points (95% CI, −6.96 to −6.04). The corresponding absolute Cohen’s dz values were 8.64 for the Constant score, 4.85 for the Taft score, 6.11 for the ASES score, and 4.06 for VAS pain. All 49 patients exceeded the prespecified Constant-score MCID threshold (100%; 95% CI, 92.7–100), whereas 47 of 49 patients exceeded the prespecified Taft-score MCID threshold (95.9%; 95% CI, 86.3–98.9). Thus, clinically meaningful improvement was nearly universal but was not identical across outcome instruments. These results are summarized in Table 5.

### 3.4. Return to Sport and Radiographic Stability

Return to unrestricted match play was recorded in all patients and occurred at a mean of 239.9 ± 43.5 days, with a median of 233 days, an interquartile range of 206–266 days, and a range of 185–363 days. Forty-four patients returned to their preinjury football level (89.8%; 95% CI, 78.2–95.6). Five patients returned to match participation but did not regain their preinjury football level. Mean injured side CC distance decreased from 22.4 ± 4.4 mm preoperatively to 9.2 ± 0.9 mm on the first postoperative radiograph and was 10.4 ± 1.6 mm at 24 months. Mean contralateral CC distance was 8.6 ± 0.9 mm. The injured-to-contralateral CCD ratio decreased from 2.62 ± 0.49 preoperatively to 1.07 ± 0.01 on the first postoperative radiograph and was 1.21 ± 0.17 at 24 months. A median 0.7 mm increase in CC distance was observed between the first postoperative radiograph and 24 months (*p* < 0.001). At 24 months, the CCD ratio ranged from 1.501 to 1.888 among the five patients, meeting the loss-of-vertical-reduction criterion, whereas the maximum ratio among the remaining 44 patients was 1.178. Consequently, post hoc thresholds ranging from >1.25 to >1.50 identified the same five patients. Interobserver agreement for CCD measurement was consistently high across all radiographic assessments. Single-measure absolute-agreement ICCs [ICC(2,1)] ranged from 0.995 to 0.998, and average-measure ICCs based on the mean of three observers [ICC(2,3)] ranged from 0.998 to 0.999. At 24 months, ICC(2,1) was 0.998 (95% CI, 0.997–0.999), and ICC(2,3) was 0.999 (95% CI, 0.999–1.000). Return-to-sport and radiographic outcomes are summarized in Table 6.

### 3.5. Exploratory Outcomes According to Reduction Status

Because only five patients met the loss-of-vertical-reduction criterion, all comparisons according to vertical reduction status were considered exploratory. Patients with loss of vertical reduction returned to unrestricted match play at a median of 344 days (IQR, 313–362), compared with 224 days (IQR, 204.8–256.5) among patients who maintained vertical reduction. The corresponding means were 332.4 ± 35.6 and 229.4 ± 29.8 days, respectively, representing an unadjusted mean difference of 103.0 days (95% CI, 59.5–146.5; Mann–Whitney U *p* < 0.001). None of the five patients with loss of vertical reduction returned to the preinjury football level, compared with all 44 patients who maintained vertical reduction (0/5 vs. 44/44; Fisher exact *p* < 0.001). The absolute risk difference was −100 percentage points (95% CI, −100 to −55.8). The vertical reduction-loss group also had lower 24-month Taft and ASES scores and higher residual VAS pain scores (all exploratory *p* < 0.001). The difference in Constant score did not reach statistical significance using the prespecified nonparametric comparison (*p* = 0.072). Patient-level chronology demonstrates that the radiographic and return-to-sport events did not have a uniform temporal sequence. Four patients first exceeded the prespecified CCD-ratio threshold at the 9-month assessment. In the remaining patient, unrestricted match play resumed on postoperative day 280, while the CCD ratio remained below the threshold at 12 months (1.249); loss of vertical reduction was first detected at 24 months (1.501). This observation further precludes interpreting the subgroup comparison as evidence that radiographic reduction loss caused delayed or incomplete return to sport. The structured retrospective dataset did not contain sufficiently detailed adjudicated variables to distinguish button migration or cut-through, suture failure, tunnel widening or osteolysis, or clavicular or coracoid fracture as the specific mechanism of vertical reduction loss in each case. Accordingly, the present study reports the timing and magnitude of radiographic vertical displacement but does not assign an unverified construct-level failure mechanism. Given the small subgroup, complete separation of the preinjury-level return outcome, unequal baseline characteristics, interval-censored radiographic detection, and the possibility of residual confounding, these findings should be interpreted as descriptive, hypothesis-generating associations rather than evidence of independent prediction or causality. Patient-level chronology is provided in Table 3.

## 4. Discussion

The principal finding of this study was that male recreational football players with acute Rockwood type IV-V acromioclavicular joint separation achieved substantial functional improvement, low residual pain, and a high rate of return to unrestricted football participation after MINAR reconstruction. In exploratory unadjusted comparisons, patients who maintained vertical reduction returned to unrestricted match play earlier and more frequently regained their preinjury football level. However, only five patients met the loss-of-vertical-reduction criterion, and patient-level chronology did not show a uniform temporal sequence between radiographic displacement and return to sport. These findings should therefore be interpreted as descriptive and hypothesis-generating rather than confirmatory, predictive, or causal.

Radiographic success and patient recovery are related but not interchangeable. Even when general shoulder function improves markedly, a recreational player may return later, at a lower level, or with residual symptoms. All patients exceeded the prespecified Constant-score MCID threshold, whereas 47 of 49 patients exceeded the Taft-score threshold. This distinction illustrates why clinically meaningful improvement in global or AC-specific scores should be considered alongside residual symptoms, vertical radiographic alignment, and sport-specific recovery.

The favorable overall clinical outcomes in this cohort are consistent with the contemporary literature reporting good function and high return-to-sport rates after operative treatment of high-grade AC joint separation [3,4,5,28,29,30,31,32,33,34]. Interpretation of the existing literature is often challenging, however, because many studies combine different Rockwood grades, acute and chronic injuries, elite and recreational athletes, manual workers, nonathletic patients, fixation constructs, and rehabilitation pathways [3,4,5,33,34]. Return to sport is also frequently reported as a binary event or as a time-based milestone, whereas sport-specific readiness and return to preinjury performance level are less consistently evaluated [5,6]. The present study reduces some of this heterogeneity by focusing on football-related acute Rockwood type IV-V injuries in male recreational players treated with the same surgical construct and institutional rehabilitation protocol.

All patients exceeded the prespecified MCID threshold for the Constant score, whereas 47 of 49 patients exceeded the corresponding Taft-score threshold. MCID achievement should not be equated with complete recovery. MCID indicates improvement beyond a clinically meaningful minimum, whereas residual symptoms, absolute final function, and return to the preinjury football level represent distinct outcome dimensions. The very large, paired effect sizes should likewise be interpreted in the context of severe functional impairment after an acute high-grade injury and the relatively limited dispersion of change scores, both of which mathematically increase Cohen’s dz. These values quantify the magnitude and consistency of within-patient recovery in this cohort, but they do not represent a comparative treatment effect or establish superiority over other techniques.

The vertical reduction-status analysis identified a clinically notable but statistically imprecise pattern. The five patients meeting the radiographic criterion returned to unrestricted match play later, had lower AC-specific and ASES scores, reported greater residual pain, and did not regain their preinjury football level. Nevertheless, the 44:5 group imbalance makes these estimates particularly vulnerable to sparse-data effects, sampling variability, and residual confounding. All five loss cases were Rockwood type V injuries and involved the dominant side, further underscoring that the observed outcomes cannot be attributed to vertical reduction status alone. These findings therefore demonstrate co-occurrence rather than establishing that vertical reduction loss caused delayed recovery or independently predicted failure to return to the preinjury level. Rehabilitation adherence, unmeasured components of injury severity, residual horizontal or rotational instability, sports exposure, and psychological readiness may have contributed to both radiographic and return-to-sport outcomes. Postoperative displacement has often been discussed as a radiographic complication with uncertain clinical importance. Some series have reported acceptable global shoulder scores despite partial CC widening, supporting the view that limited loss of reduction may be clinically tolerable in selected patients [35,36,37,38,39]. The present findings suggest that the potential relationship between maintenance of vertical reduction and sport-specific recovery warrants evaluation in larger cohorts.

The present radiographic endpoint must also be interpreted anatomically. Serial CCD measurement primarily reflects vertical CC alignment and does not provide a complete assessment of three-dimensional AC joint stability. The CC ligament complex contributes importantly to vertical stability, whereas the AC capsuloligamentous structures contribute to horizontal and rotational control [40,41,42,43,44,45,46]. Because the AC capsule was not repaired and dynamic horizontal translation was not quantified, maintenance of vertical reduction cannot be assumed to indicate restoration or maintenance of horizontal stability. This limitation is particularly relevant to Rockwood type IV injuries, in which posterior displacement is a defining feature. In recreational football, the shoulder may be exposed to contact, unexpected collisions, falls, protective upper-limb loading, and rapid balance reactions, but the present study did not directly assess scapular kinematics, dynamic horizontal stability, or sport-specific biomechanics. Any biomechanical explanation for the observed clinical pattern therefore remains explanatory rather than demonstrated.

These findings also support a multidimensional approach to return-to-sport assessment after AC reconstruction. Recent reviews have shown that return-to-play criteria after operative AC separation are most often time-based, with a limited reporting of objective strength symmetry, dynamic stability, functional testing, sport-specific performance, and psychological readiness [5,6,47]. In this cohort, all patients eventually resumed unrestricted match participation, but not all returned to their preinjury football level. Because return was largely self-directed and formal team-based clearance was unavailable, the recorded outcome should be interpreted as return to participation rather than standardized medical clearance. Serial radiographic assessment of vertical CC alignment may provide complementary follow-up information, but it should not be used as an isolated return-to-sport criterion. Future studies should combine physical recovery criteria with validated measures of fear of reinjury and psychological readiness.

The recreational football population merits separate consideration. The sports-medicine literature often emphasizes elite and professional athletes, but recreational players represent a large proportion of real-world orthopedic practice. This group may differ from professional athletes not necessarily in AC injury severity but in training structure, access to supervised rehabilitation, neuromuscular preparedness, load management, and medical oversight [2,10,11,12,13]. Professional players typically recover within structured systems that include physiotherapists, strength and conditioning staff, team physicians, and progressive field-based return protocols. Recreational football players may return in less controlled environments, often guided by symptoms, social expectations, or self-assessment. The present study does not claim that recreational football players sustain more severe AC separations than professional athletes; current AC-specific evidence does not support such a conclusion. A more defensible interpretation is that recreational athletes represent a common and clinically relevant subgroup whose postoperative recovery and return-to-sport pathway should be evaluated directly.

The clinical implications of this study extend beyond assessment of the MINAR technique itself. For the surgeon, anatomic alignment at the end of the operation represents successful reconstruction. For the patient, recovery is complete only when the shoulder girdle can tolerate the unrestricted biomechanical demands of sport. Serial postoperative radiographs should therefore be viewed as documentation of implant position and maintenance of the vertical CC relationship during biological healing, not as a stand-alone measure of overall AC joint stability or a return-to-sport clearance test [15,16,17,18,36,37,38,39]. In patients with progressive displacement, closer follow-up, a more cautious return-to-sport timeline, and individualized rehabilitation emphasizing scapular control, strength restoration, and sport-specific readiness may be reasonable. These suggestions should not be interpreted as a definitive change in clinical algorithms based on a single-center retrospective study, but they may help frame reduction maintenance as a complementary marker of recovery quality. These considerations represent cautious clinical implications rather than evidence-based management recommendations and require prospective evaluation.

The use of transosseous tunnels also introduces a technique-specific consideration. MINAR requires osseous passage through the coracoid and the clavicle, and tunnel-dependent constructs may be susceptible to failure mechanisms such as tunnel enlargement, implant cut-through, or fracture through structurally weakened bone. Ranson et al. described a tunnel-less modification of an all-suture tape cerclage technique in which the tape is passed around the clavicle without cortical violation, with the aim of avoiding drill-tunnel-related structural compromise [48]. This represents a relevant technical tradeoff when considering CC stabilization strategies. However, the present cohort did not include a tunnel-less comparator and cannot establish differences in fracture, cut-through, maintenance of reduction, fixation failure, or clinical outcome rates between techniques.

This study has several strengths. The cohort was homogeneous with respect to sex, sports type, injury mechanism, acute timing, surgical timing, experienced surgical team, implant platform, and department-supervised rehabilitation setting. Clinical outcomes were assessed using established shoulder and AC-specific scores. Return to sport was analyzed not only as a yes/no event but also according to unrestricted match-level return and return to preinjury football level. Radiographic evaluation of vertical alignment was based on objective CC distance measurements rather than subjective interpretation alone. These features reduce some of the heterogeneity that commonly limits AC joint outcome studies.

Several limitations should be considered. This study was based on retrospective review of routine clinical records; therefore, the observed associations should not be interpreted as evidence of causality. Only five patients met the prespecified criterion for loss of vertical reduction, producing a marked 44:5 group imbalance. Consequently, reduction-status estimates and *p*-values are vulnerable to sparse-data effects, sampling variability, and residual confounding and should be interpreted descriptively. All subgroup analyses were unadjusted, and no prediction model was fitted. This study was performed at a single center by the same experienced surgical team, which increased technical consistency but may limit external generalizability. No universally accepted radiographic definition of reduction loss exists. The present study used a prespecified injured-to-contralateral CCD ratio greater than 1.50 and additionally reported continuous CCD ratios and alternative-threshold sensitivity analyses. Although interobserver reliability was high, dichotomization of a continuous radiographic measurement may still produce classification uncertainty, particularly for values close to the selected threshold. Radiographic follow-up assessed vertical CC alignment rather than complete three-dimensional AC joint stability. Dynamic horizontal and rotational translation were not quantified, and the AC capsule was not repaired; therefore, this study cannot determine whether residual horizontal instability contributed to clinical or sport-specific outcomes. Static supine CT could identify posterior clavicular displacement at diagnosis but could not quantify dynamic horizontal translation under functional loading. MRI was not routinely obtained because the diagnosis and operative indication were established clinically and radiographically; therefore, clinically silent associated intra-articular lesions could not be completely excluded. Although rehabilitation was delivered within the same physiotherapy department, progression was individualized, quantitative session-level adherence was not consistently available, and the exact duration of sling use was not uniformly retrievable from the analyzable retrospective dataset. Return-to-sport dates were based on patient-reported participation documented during follow-up, and formal team-based clearance was generally unavailable. The structured retrospective dataset also did not permit reliable adjudication of the construct-level mechanism underlying each case of loss of vertical reduction, including button migration or cut-through, suture failure, tunnel widening or osteolysis, or clavicular/coracoid fracture. Accordingly, mechanism-specific conclusions were not drawn. Psychological readiness, fear of reinjury, and sport-related confidence were not assessed using a validated instrument; consequently, their contribution to the timing or level of return could not be determined. Finally, because the cohort consisted only of male recreational small-sided football players, the results may not be directly generalizable to female athletes, professional players, chronic AC instability, other sports, or other reconstructive techniques.

Future prospective multicenter studies should evaluate whether postoperative maintenance of vertical reduction is associated with return-to-sport timing and recovery quality across different surgical techniques, sports, and competition levels. Imaging protocols should ideally assess vertical, horizontal, and rotational stability because AC joint function is three-dimensional [16,17,44]. Follow-up should incorporate objective functional metrics, including strength symmetry, scapular kinematics, sport-specific closed-chain tests, and validated psychological-readiness measures alongside patient-reported outcomes [34,45,46]. It would also be valuable to determine whether targeted rehabilitation or modified return-to-play timing in patients with early reduction loss can improve sport-specific outcomes. Future studies should enroll a sufficient number of reduction-loss events to permit appropriately powered multivariable analyses and should assess reduction status as both a continuous CCD ratio and a prespecified categorical outcome.

## 5. Conclusions

Male recreational football players with acute Rockwood type IV-V acromioclavicular joint separation demonstrated substantial functional improvement, minimal residual pain, and high rates of return to football after MINAR reconstruction. In exploratory unadjusted comparisons, the five patients meeting the loss-of-vertical-reduction criterion returned to unrestricted match play later and did not regain their preinjury football level, whereas all patients who maintained vertical reduction returned to their previous level. However, the small and imbalanced subgroup, potential residual confounding, and nonuniform temporal relationship between radiographic threshold crossing and return to sport preclude causal or predictive inference. These observations should therefore be regarded as hypothesis-generating. Larger prospective studies incorporating vertical, horizontal, and rotational stability measures are required to determine the independent clinical relevance of postoperative maintenance of vertical reduction.

## Figures and Tables

**Table 1:** Cohort characteristics and perioperative variables.

Variable	Value
Patients, n	49
Age, years	35.1 ± 6.2 (25–45)
BMI, kg/m^2^	27.9 ± 5.2 (19.8–42.7)
Current smoker	27 (55.1%)
Right-sided injury	32 (65.3%)
Dominant side injured	31 (63.3%)
Rockwood type IV	8 (16.3%)
Rockwood type V	41 (83.7%)
Injury-to-surgery interval, days	2.8 ± 1.1 (1–6)
Follow-up, months	48.6 ± 14.5 (23.7–76.5)
Reoperation	0 (0%)
Infection	0 (0%)
Hardware irritation	0 (0%)
Radiographic loss of vertical reduction	5 (10.2%; 95% CI, 4.4–21.8)

Data are presented as means ± SDs (ranges) or n (%). BMI, body mass index; CI, confidence interval; SD, standard deviation. Radiographic loss of vertical reduction was defined as a 24-month injured-to-contralateral CCD ratio greater than 1.50.

**Table 2:** Baseline characteristics according to maintenance of vertical reduction.

Variable	Maintained Vertical Reduction (n = 44)	Loss of Vertical Reduction (n = 5)
Age, years	34.5 ± 6.1	40.4 ± 3.5
BMI, kg/m^2^	28.0 ± 5.3	27.3 ± 4.5
Current smoker, n/N (%)	24/44 (54.5%)	3/5 (60.0%)
Dominant side injured, n/N (%)	26/44 (59.1%)	5/5 (100%)
Rockwood type IV, n/N (%)	8/44 (18.2%)	0/5 (0%)
Rockwood type V, n/N (%)	36/44 (81.8%)	5/5 (100%)
Right-sided injury, n/N (%)	27/44 (61.4%)	5/5 (100%)
Injury-to-surgery interval, days	2.8 ± 1.1	3.4 ± 1.5
Follow-up, months	48.9 ± 14.9	45.9 ± 12.1

Data are presented as means ± SDs or n/N (%). Reduction status was defined using the prespecified 24-month injured-to-contralateral CCD-ratio threshold of >1.50. Because only five patients met the loss-of-vertical-reduction criterion, baseline variables are presented descriptively, and no between-group hypothesis tests were performed. Quantitative rehabilitation-adherence data were not available for retrospective analysis. BMI, body mass index; CCD, coracoclavicular distance; SD, standard deviation.

**Table 3:** Patient-level chronology of loss of vertical reduction and return-to-sport outcomes.

Case	Rockwood	Last Scheduled Assessment Below 1.50	First Assessment > 1.50	24-Month CCD Ratio	Unrestricted Match Return, Days	Return to Preinjury Level
F1	V	6 m: 1.092	9 m: 1.587	1.573	344	No
F2	V	6 m: 1.094	9 m: 1.718	1.755	362	No
F3	V	6 m: 1.064	9 m: 1.845	1.888	363	No
F4	V	12 m: 1.249	24 m: 1.501	1.501	280	No
F5	V	6 m: 1.082	9 m: 1.659	1.687	313	No

Cases are deidentified. All five were Rockwood type V injuries. “First assessment > 1.50” refers to the first scheduled radiographic examination at which the injured-to-contralateral CCD ratio exceeded the prespecified threshold and does not establish the exact biological time of onset. Specific mechanisms such as button migration or cut-through, suture failure, tunnel widening or osteolysis, and clavicular or coracoid fracture were not encoded as adjudicated variables in the structured study dataset.

**Table 4:** Longitudinal clinical outcomes from preoperative assessment to 24 months.

Outcome	Preoperative	3 Months	6 Months	12 Months	24 Months	Overall *p*-Value
Constant score	29.9 ± 6.0	89.9 ± 6.4	93.4 ± 4.6	93.8 ± 3.8	94.5 ± 3.8	<0.001
Taft score	4.7 ± 0.7	8.6 ± 0.2	9.9 ± 0.8	10.4 ± 0.8	10.8 ± 0.8	<0.001
ASES score	45.8 ± 3.5	75.3 ± 2.2	88.0 ± 1.6	88.4 ± 5.6	89.1 ± 5.6	<0.001
VAS pain	6.8 ± 1.5	4.2 ± 1.1	1.7 ± 1.2	0.6 ± 0.8	0.3 ± 0.5	<0.001

Values are means ± SDs. Overall *p*-values were obtained using the Friedman test. The original 12-point Taft score was used. ASES, American Shoulder and Elbow Surgeons; SD, standard deviation; VAS, visual analog scale.

**Table 5:** Preoperative-to-24-month changes, precision, and clinical relevance.

Outcome	Change, Mean ± SD (95% CI)	*p*-Value	Cohen’s dz	MCID Responders, n/N (%)
Constant score	64.60 ± 7.47 (62.45 to 66.75)	<0.001	8.64	49/49 (100%; 95% CI, 92.7–100)
Taft score	6.11 ± 1.26 (5.74 to 6.47)	<0.001	4.85	47/49 (95.9%; 95% CI, 86.3–98.9)
ASES score	43.32 ± 7.09 (41.28 to 45.35)	<0.001	6.11	—
VAS pain	−6.50 ± 1.60 (−6.96 to −6.04)	<0.001	4.06	—

For Constant, Taft, and ASES scores, positive changes indicate improvement; for VAS pain, negative changes indicate improvement. Absolute Cohen’s dz values are shown. MCID responder analysis was prespecified for the Constant and Taft scores using thresholds of 16.6 and 2.9 points, respectively. ASES, American Shoulder and Elbow Surgeons; CI, confidence interval; MCID, minimal clinically important difference; SD, standard deviation; VAS, visual analog scale.

**Table 6:** Return-to-sport, 24-month clinical, and radiographic outcomes according to vertical reduction status.

Outcome	Overall Cohort (n = 49)	Maintained Vertical Reduction (n = 44)	Loss of Vertical Reduction (n = 5)	*p*-Value
Return to unrestricted match play, days	239.9 ± 43.5	229.4 ± 29.8	332.4 ± 35.6	<0.001
Return to preinjury football level, n/N (%)	44/49 (89.8%)	44/44 (100%)	0/5 (0%)	<0.001
Constant score at 24 months	94.5 ± 3.8	94.9 ± 3.8	91.3 ± 1.8	0.072
Taft score at 24 months	10.8 ± 0.8	11.0 ± 0.5	8.8 ± 0.6	<0.001
ASES score at 24 months	89.1 ± 5.6	90.7 ± 2.4	75.4 ± 6.9	<0.001
VAS pain at 24 months	0.3 ± 0.5	0.2 ± 0.4	1.2 ± 0.4	<0.001
Contralateral CC distance, mm	8.6 ± 0.9	—	—	—
Injured-side CC distance, preoperative, mm	22.4 ± 4.4	—	—	—
Injured-side CC distance, first postoperative radiograph, mm	9.2 ± 0.9	—	—	—
Injured-side CC distance at 24 months, mm	10.4 ± 1.6	—	—	—

Continuous variables are presented as means ± SDs and categorical variables as n/N (%). Reduction status refers specifically to maintenance of vertical reduction based on the prespecified 24-month injured-to-contralateral CCD-ratio threshold of >1.50. Group comparisons used the Mann–Whitney U test or Fisher exact test, as appropriate. Because only five patients met the loss-of-vertical-reduction criterion, these comparisons are exploratory, and the *p*-values should be interpreted descriptively. The unadjusted mean difference in time to unrestricted match play was 103.0 days (95% CI, 59.5–146.5), and the absolute risk difference for return to the preinjury football level, calculated as loss minus maintained vertical reduction, was −100 percentage points (95% CI, −100 to −55.8). ASES, American Shoulder and Elbow Surgeons; CC, coracoclavicular; CI, confidence interval; SD, standard deviation; VAS, visual analog scale.

## Data Availability

The datasets generated and analyzed during the current study are not publicly available due to institutional privacy and ethical restrictions but are available from the corresponding author upon reasonable request and with appropriate institutional approval.

## Abbreviations

AC	acromioclavicular
AP	anteroposterior
ASES	American Shoulder and Elbow Surgeons
BMI	body mass index
CC	coracoclavicular
CCD	coracoclavicular distance
CI	confidence interval
CT	computed tomography
ICC	intraclass correlation coefficient
IQR	interquartile range
MCID	minimal clinically important difference
MINAR	minimally invasive acromioclavicular/coracoclavicular reconstruction
MRI	magnetic resonance imaging
PACS	picture archiving and communication system
SD	standard deviation
STROBE	Strengthening the Reporting of Observational Studies in Epidemiology
VAS	visual analog scale
